# Ascending aorta backward flow parameters estimated from phase-contrast cardiovascular magnetic resonance data: new indices of arterial aging

**DOI:** 10.1186/1532-429X-14-S1-P128

**Published:** 2012-02-01

**Authors:** Zoubir M Bensalah, Emilie Bollache, Nadjia Kachenoura, Alain De Cesare, Alban Redheuil, Elie Mousseaux

**Affiliations:** 1 INSERM 678 / UPMC, Hôpital Pitié-Salpêtrière, Paris, France; 2 Radiology, Hôpital Ambroise Paré, Paris, France; 3 Radiology, Hôpital Européen Georges Pompidou, Paris, France

## Summary

Our purpose was to estimate volume and flow rate parameters related to the backward flow in the ascending aorta (AA) using phase-contrast cardiovascular magnetic resonance (PC-CMR) and to evaluate their relationships with age and with well established arterial stiffness indices including wave reflection parameters in an asymptomatic group without overt cardiovascular disease.

## Background

Aging is a major risk factor for cardiovascular disease and is a prime determinant of aortic stiffness. Backward flow has been previously described using PC-CMR, but only few studies focused on the comparison between backward flow parameters and arterial stiffness indices.

## Methods

We studied 80 asymptomatic subjects without overt cardiovascular disease and free of significant aortic regurgitation (50 men, age: 42±17 years) who underwent cine and PC-CMR imaging of the thoracic aorta (1.5T) as well as carotid-femoral tonometry (PulsePen). Tonometry provided carotid-femoral pulse wave velocity (PWVCF), as well as reflection parameters such as carotid augmentation index (AIx) and Ti (time between pressure upstroke and the systolic inflection point). CMR images were analyzed using a custom software (ArtFun), which provides an automated segmentation of the AA and descending aorta throughout the cardiac cycle and a semi-automated estimation of aortic functional parameters, such as conventional aortic stiffness indices (aortic arch pulse wave velocity (PWVAo) and AA distensibility), as well as parameters related to the backward flow including global backward volume VB and backward flow rate peak QBmax, both calculated from backward flow rate curves (Figure). Furthermore, VF and QFmax parameters were calculated from the forward flow rate curves.

**Figure 1:**
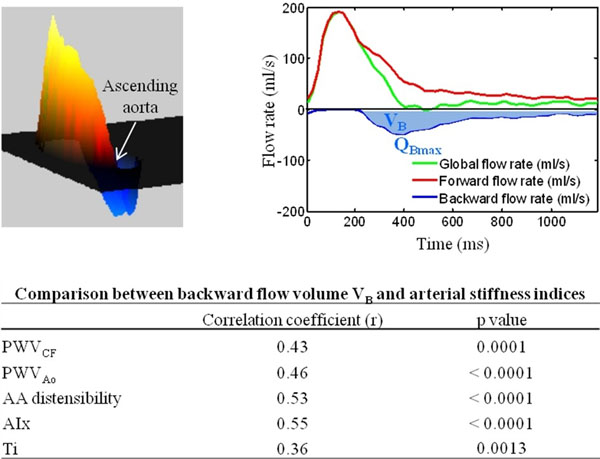
PC CMR data of a 66 year-old female subject. Left: AA forward (hot tones) and backward (cold tones) through-plane velocity profile in a systolic phase. Right: the corresponding flow rate curves and the estimated backward flow indices.

## Results

Mean values were: 70.2 ± 22.4 ml for the aortic systolic forward volume and 13.2 ± 9.4 ml for the backward volume VB. Mean value of QBmax was 55.8 ± 27.5 ml/sec. While non-significant correlations were found for the comparisons against age of forward flow parameters VF (r=0.25, p=0.03) and QFmax (r=0.23, p=0.025), significant correlations were obtained with age for backward flow parameters VB (r=0.63, p<0.0001) and QBmax (r=0.55, p<0.0001). VB also significantly correlated with arterial stiffness indices (Table). Similar results were obtained when considering QBmax.

## Conclusions

AA backward flow parameters estimated from PC-CMR significantly correlated with age as well as with conventional arterial stiffness indices. These backward flow parameters, which can be easily estimated using an automated analysis of PC-CMR data, could, when combined with parameters of left ventricular (LV) function, help to increase the understanding of arterial aging and LV-arterial coupling mechanisms.

## Funding

None.

